# Regulation of STEP_61_ and tyrosine-phosphorylation of NMDA and AMPA receptors during homeostatic synaptic plasticity

**DOI:** 10.1186/s13041-015-0148-4

**Published:** 2015-09-22

**Authors:** Sung-Soo Jang, Sara E. Royston, Jian Xu, John P. Cavaretta, Max O. Vest, Kwan Young Lee, Seungbae Lee, Han Gil Jeong, Paul J. Lombroso, Hee Jung Chung

**Affiliations:** Department of Molecular and Integrative Physiology, University of Illinois at Urbana-Champaign, 407 South Goodwin Avenue, 524 Burrill Hall, Urbana, IL 61801, USA; Neuroscience Program, University of Illinois at Urbana-Champaign, Urbana, IL 61801, USA; Medical Scholars Program, University of Illinois at Urbana-Champaign, Urbana, IL 61801, USA; Child Study Center, New Haven, CT 06510, USA; Department of Neurobiology and Psychiatry, Yale University School of Medicine, New Haven, CT 06510, USA

**Keywords:** STEP, GluN2B, GluA2, Tyrosine phosphorylation, Tetrodotoxin, Bicuculline, Hippocampal neurons, Homeostatic plasticity, Synaptic scaling

## Abstract

**Background:**

Sustained changes in network activity cause homeostatic synaptic plasticity in part by altering the postsynaptic accumulation of N-methyl-D-aspartate receptors (NMDAR) and α-amino-3-hydroxyle-5-methyl-4-isoxazolepropionic acid receptors (AMPAR), which are primary mediators of excitatory synaptic transmission. A key trafficking modulator of NMDAR and AMPAR is STriatal-Enriched protein tyrosine Phosphatase (STEP_61_) that opposes synaptic strengthening through dephosphorylation of NMDAR subunit GluN2B and AMPAR subunit GluA2. However, the role of STEP_61_ in homeostatic synaptic plasticity is unknown.

**Findings:**

We demonstrate here that prolonged activity blockade leads to synaptic scaling, and a concurrent decrease in STEP_61_ level and activity in rat dissociated hippocampal cultured neurons. Consistent with STEP_61_ reduction, prolonged activity blockade enhances the tyrosine phosphorylation of GluN2B and GluA2 whereas increasing STEP_61_ activity blocks this regulation and synaptic scaling. Conversely, prolonged activity enhancement increases STEP_61_ level and activity, and reduces the tyrosine phosphorylation and level of GluN2B as well as GluA2 expression in a STEP_61_–dependent manner.

**Conclusions:**

Given that STEP_61_-mediated dephosphorylation of GluN2B and GluA2 leads to their internalization, our results collectively suggest that activity-dependent regulation of STEP_61_ and its substrates GluN2B and GluA2 may contribute to homeostatic stabilization of excitatory synapses.

**Electronic supplementary material:**

The online version of this article (doi:10.1186/s13041-015-0148-4) contains supplementary material, which is available to authorized users.

## Findings

### Background

In response to sustained changes in neuronal activity, homeostatic synaptic plasticity maintains synaptic strength and flexibility within physiological limit. This plasticity is expressed in part by dynamic changes in the postsynaptic levels of NMDARs and AMPARs that mediate excitatory synaptic transmission [1]. A key trafficking modulator of both NMDAR and AMPAR is STEP_61_, a protein tyrosine (Tyr) phosphatase in the central nervous system that has two main alternatively spliced forms, the cytosolic STEP_46_ and the membrane-associated STEP_61_ [2]. Tightly associated with the postsynaptic density, STEP_61_ regulates the Tyr phosphorylation and surface density of NMDARs and AMPARs [3–6]. This regulation contributes to Hebbian long-term potentiation [4, 7] and several neuropsychiatric disorders most notably Alzheimer’s disease [4] and Fragile X-syndrome [2].

We have previously identified mRNA transcripts whose expressions are regulated by prolonged activity perturbation [8] due to a critical role of transcription in homeostatic synaptic plasticity [9, 10]. Of these activity-regulated transcripts, we identified *PTPN5* that encodes STEP [8]. The present study investigated whether STEP_61_ contributes to homeostatic synaptic plasticity.

## Results and discussion

### Prolonged alterations of hippocampal network activity regulate STEP_61_ level and activity

**Fig. 1:**
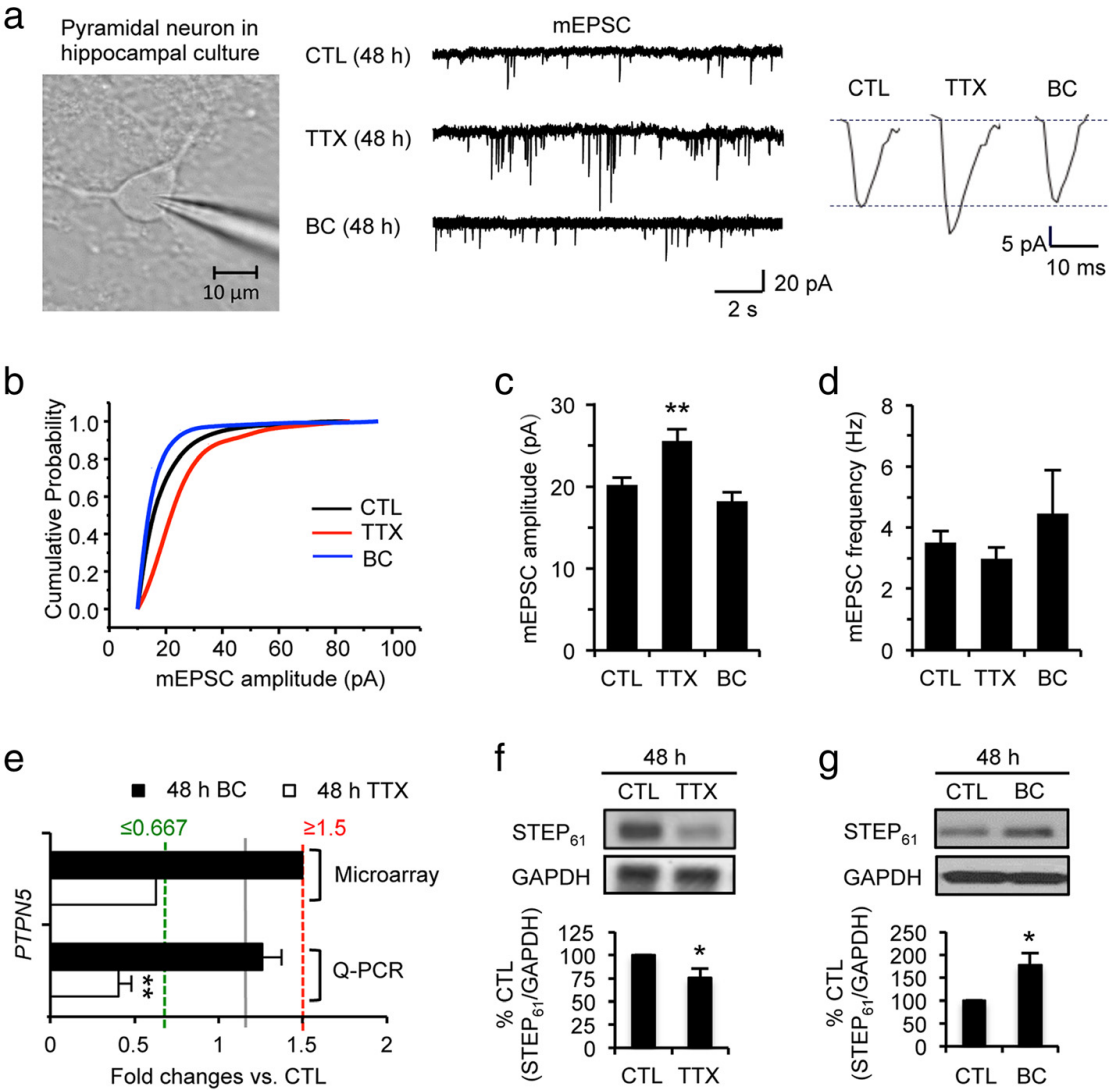
Prolonged alterations of hippocampal network activity induce homeostatic synaptic plasticity and regulate STEP_61_ level. **a**–**d** Whole-cell patch clamp recording of miniature excitatory postsynaptic currents (mEPSCs) from rat dissociated hippocampal neurons cultured in high density that were treated for 48 h with vehicle control (CTL, 0.1 % H_2_O), TTX (1 μM), or BC (20 μM) at 12–14 days in vitro. **a** Representative traces of mEPSCs. **b** Normalized cumulative fraction of the mEPSC amplitudes. **c**–**d** Summary plots of average mEPSC amplitudes (**c**) and frequencies (**d**) for CTL (*n* = 19), TTX (*n* = 20), or BC (*n* = 16). TTX treatment for 48 h induced synaptic scaling whereas 48 h BC treatment had no effect. **e** Microarray (*n* = 4) and QPCR (*n* = 5) analyses revealed that 48 h application of TTX but not BC reduced the expression of *PTPN5*, which encodes STEP. **f**–**g** Immunoblot analysis of STEP_61_ following 48 h administration of CTL, TTX (F), BC (**g**) (*n* = 9 per treatment). Data shown represent the mean ± SEM (*p < 0.05; **p < 0.01)

Prolonged blockade of network activity for 48 h with the sodium channel blocker tetrodotoxin (TTX) induced synaptic scaling in dissociated hippocampal cultured neurons as demonstrated previously [11, 12] (Fig. 1a–d), and reduced STEP_61_ mRNA and protein expression compared to CTL treatment (Fig. 1e, f). Conversely, prolonged activity enhancement for 48 h using the GABA_A_ receptor antagonist bicuculline (BC) increased STEP_61_ protein level (Fig. 1g), but did not alter its mRNA level and the miniature excitatory postsynaptic current (mEPSC) (Fig. 1a–e).

**Fig. 2:**
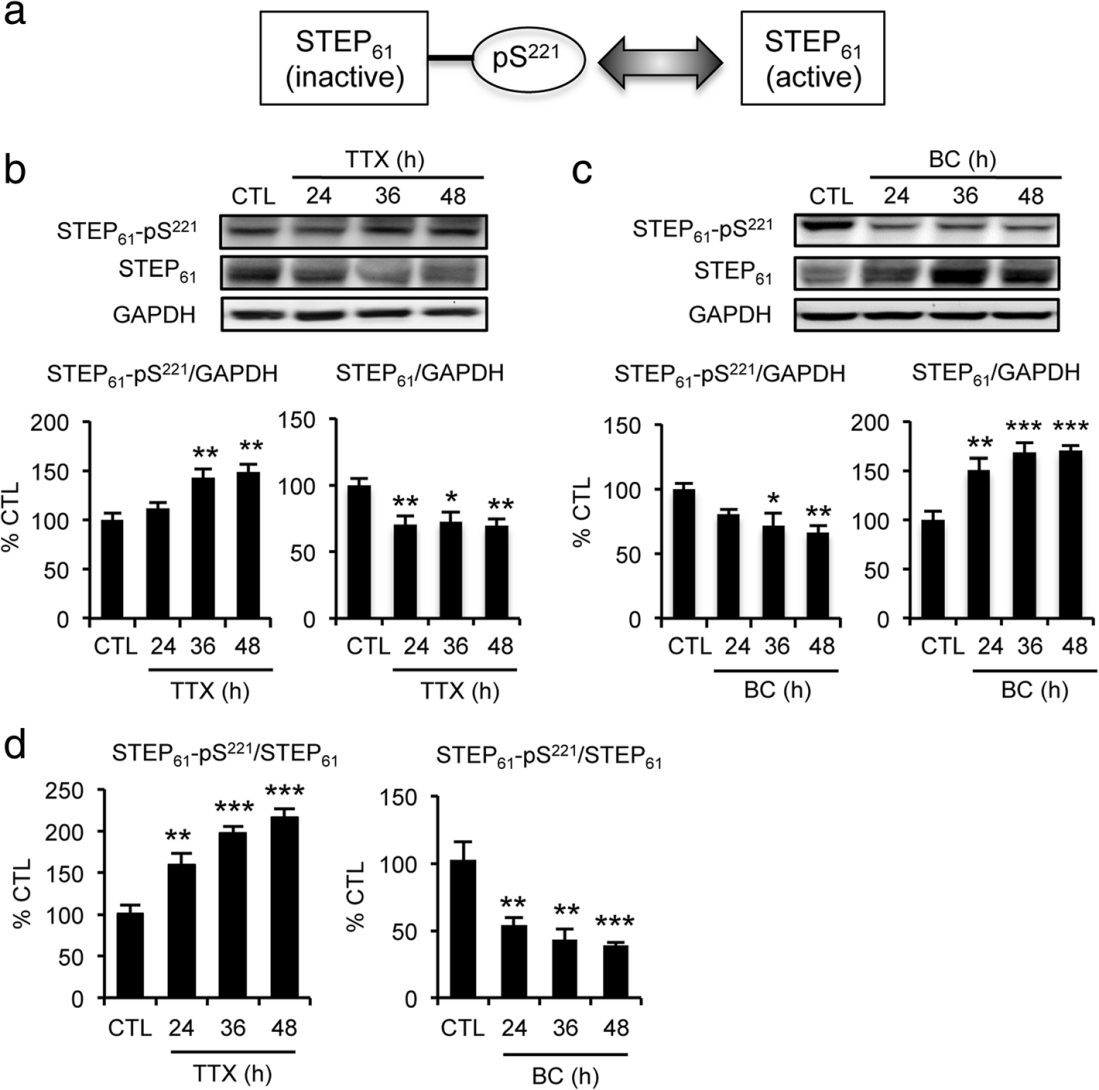
Prolonged alterations of hippocampal network activity regulate STEP_61_ activity. **a** A schematic depicting the regulation of STEP_61_ activity by its phosphorylation at Ser^221^ within its kinase-interactive motif, a binding site for all STEP substrates. **b**–**d** Immunoblot analysis of STEP_61_ and Ser^221^–phosphorylated STEP_61_ (STEP_61_-pS^221^) in hippocampal cultured neurons following CTL, TTX, or BC treatment for 24–48 h (*n* = 3 per treatment). **b**, **d** Prolonged TTX treatment reduced STEP_61_ protein level and activity. **c**, **d** Prolonged BC treatment enhanced STEP_61_ protein level and activity. **d** The relative phosphorylation state of STEP_61_ as calculated by the ratio of STEP_61_-pS^221^ level over total STEP_61_ level. Data shown represent the mean ± SEM (*p < 0.05; **p < 0.01; ***p < 0.005)

To test whether prolonged TTX or BC treatment affects STEP_61_ activity, we examined the phosphorylation of STEP_61_ at Ser^221^ within its kinase-interactive motif domain, which prevents STEP_61_ interaction with all known substrates (Fig. 2a) [13]. TTX treatment for 36–48 h enhanced Ser^221^–phosphorylation of STEP_61_, indicating decreased STEP_61_ activity (Fig. 2b, d). In contrast, 36–48 h BC treatment reduced Ser^221^–phosphorylation, indicating increased STEP_61_ activity (Fig. 2c, d).

### Prolonged alterations of hippocampal network activity regulate Tyr-phosphorylation of GluN2B and GluA2 in a STEP_61_-dependent manner

**Fig. 3:**
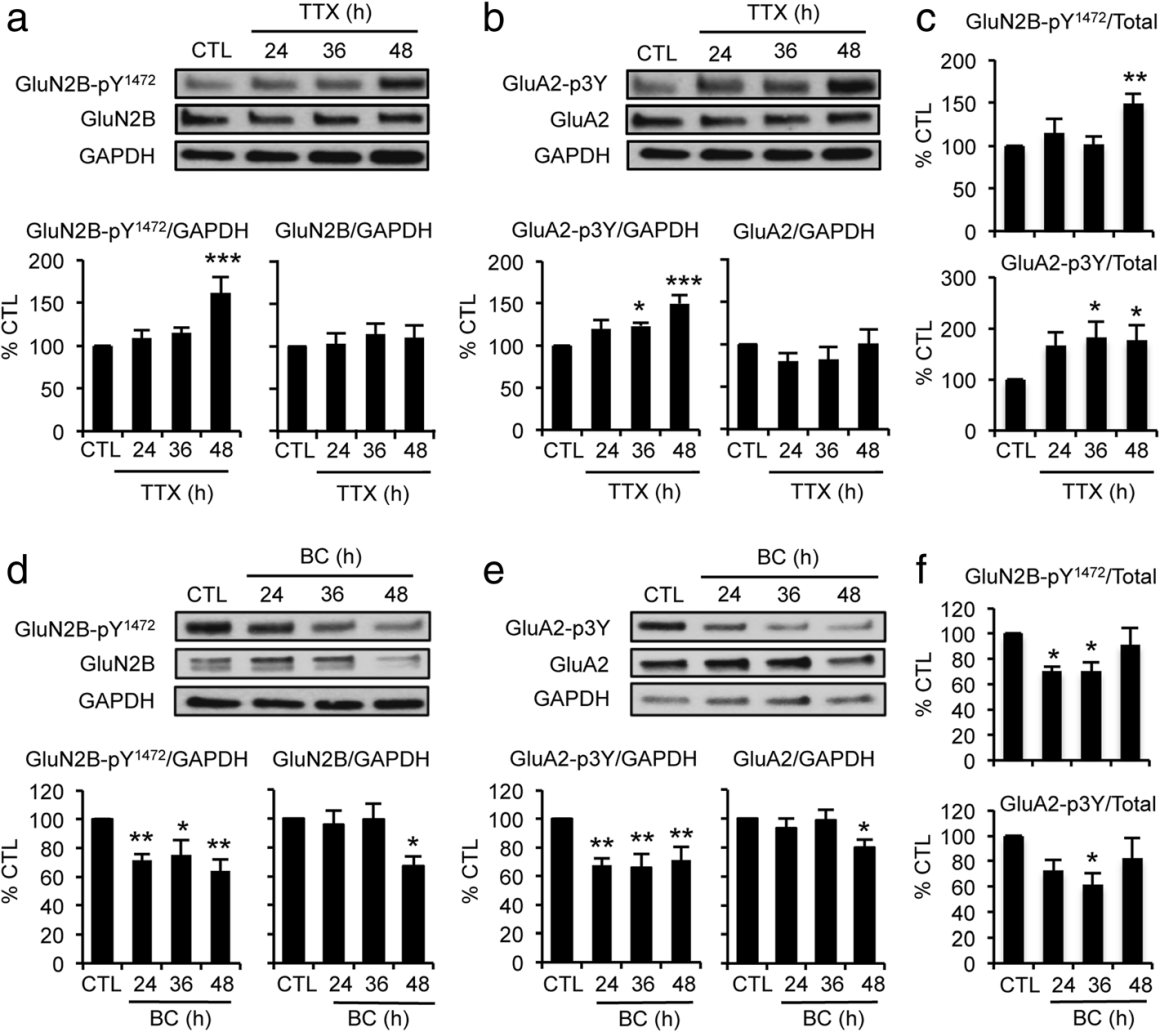
Prolonged alterations of hippocampal network activity regulate Tyr-phosphorylation and levels of GluN2B and GluA2. Immunoblot analysis of hippocampal cultured neurons that were treated for 48 h with CTL, 24–48 h TTX (**a**–**c**), or 24–48 h BC treatment (**d**–**f**) (*n* = 6 per treatment). **a**–**c** Prolonged TTX treatment increased the level of Tyr^1472^–phosphorylated GluN2B (GluN2B-pY^1472^) (**a**) and the level of GluA2 that were phosphorylated at Tyr ^869^, Tyr ^873^, and Tyr ^876^ (3Tyr) (GluA2-p3Y) (**b**). **d**–**f** Prolonged BC treatment decreased the levels of GluN2B-pY^1472^ (**d**) and GluA2-p3Y (**e**). Total GluN2B and GluA2 levels were reduced at 48 h BC application. **c**, **f** The relative phosphorylation state of GluN2B and GluA2 as calculated by the ratio of phosphorylated proteins over total proteins followed by normalization to GAPDH. Data shown represent the mean ± SEM (*p < 0.05; **p < 0.01)

STEP_61_ dephosphorylates the NMDAR subunit GluN2B at Tyr^1472^ [5, 6] and reduces Tyr-phosphorylation of the AMPAR subunit GluA2 following group 1 metabotropic glutamate receptor (mGluR) stimulation [3]. Although the specific Tyr residues on GluA2 regulated by STEP_61_ are unknown, the GluA2 phosphorylation state at Tyr^869^, Tyr^873^, and Tyr^876^ (3Tyr) regulates AMPAR trafficking [14]. To determine if the TTX- or BC-induced changes in STEP_61_ alter Tyr-phosphorylation of GluN2B and GluA2, we performed immunoblot analyses using specific antibodies to phosphorylated Tyr^1472^ of GluN2B [6] and phosphorylated 3Tyr of GluA2 [14] (Fig. 3).

Consistent with the TTX-induced decrease in STEP_61_ level and activity (Fig. 2b, d), prolonged TTX treatment increased the levels of Tyr^1472^-phosphorylated GluN2B (GluN2B-pY^1472^) and 3Tyr-phopshorylated GluA2 (GluA2-p3Y) compared to CTL treatment without affecting their total protein expression (Fig. 3a–c). In contrast, BC treatment for 24–48 h decreased the levels of GluN2B-pY^1472^ and GluA2-p3Y (Fig. 3d–f), concurrently with an increase in STEP_61_ level and activity (Fig. 2c, d). Interestingly, total levels of GluN2B and GluA2 were reduced by 48 h BC application (Fig. 3d, e).

**Fig. 4:**
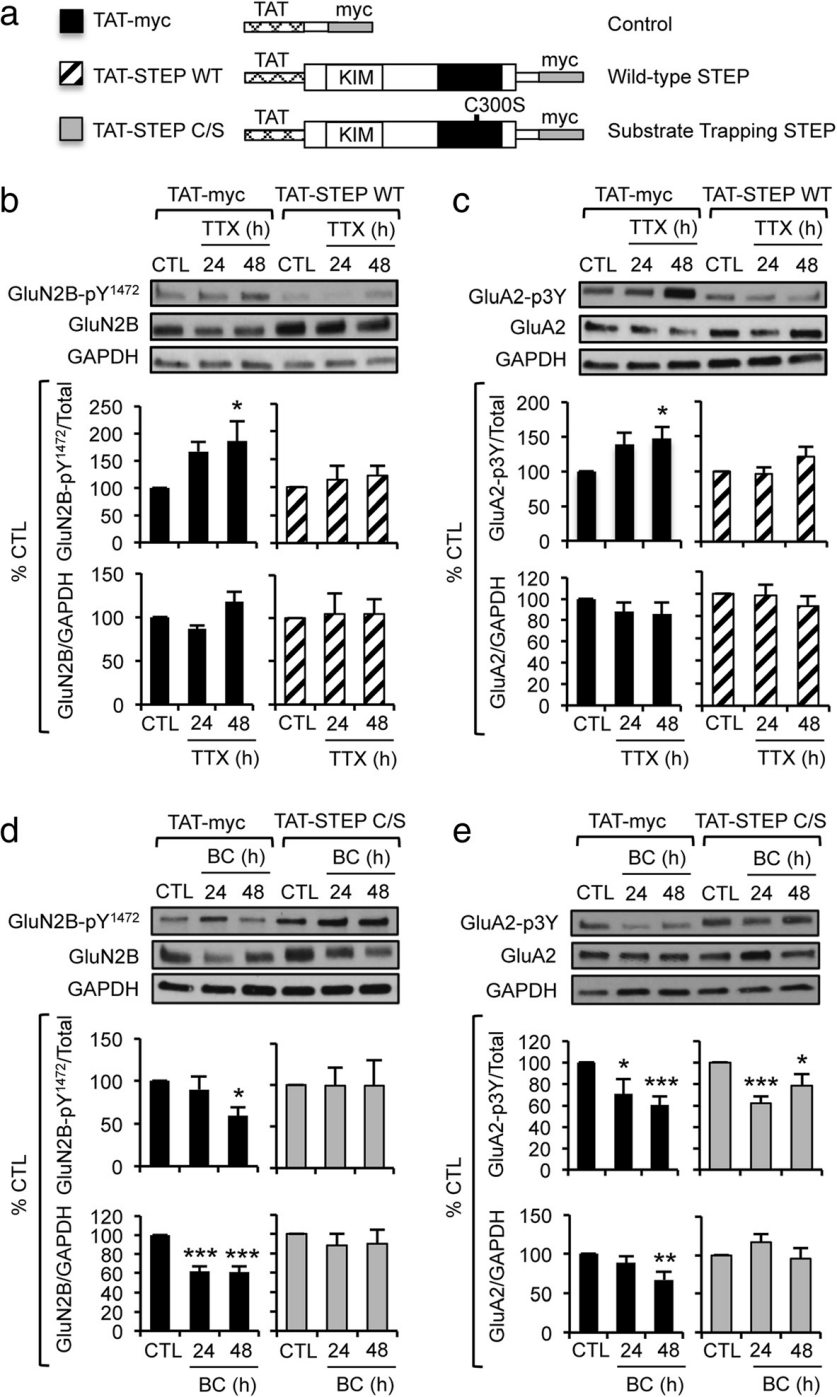
TAT-STEP WT or C/S blocks the activity-induced bidirectional changes in GluN2B and GluA2. **a** A simplified schematic (not to scale) of cell-permeable TAT-STEP molecular tools. **b**–**e** Immunoblot analysis of hippocampal cultured neurons that were treated for CTL, TTX (**b**–**c**), or BC (**d**–**e**) for 24 h and 48 h (*n* = 8 per treatment). Prior to neuronal lysate preparation, neurons were preincubated for 30 min with TAT-myc, TAT-STEP WT, or TAT-STEP C/S proteins. **b**–**c** TAT-STEP WT blocked the TTX-induced increase in Tyr^1472^–phosphorylation of GluN2B (GluN2B-pY^1472^) (**b**) and 3Tyr-phosphorylation of GluA2 (GluA2-p3Y) (**c**). **d**–**e** TAT-STEP C/S blocked the BC-induced reduction in Tyr^1472^–phosphorylation and level of GluN2B (**d**) as well as GluA2 level but not 3Tyr–phosphorylation of GluA2 (**e**). Data shown represent the mean ± SEM following normalization to CTL values in the presence of TAT-myc (black bars), TAT-STEP WT (striped bars), and TAT-STEP C/S (gray bars) (*p < 0.05; **p < 0.01; ***p < 0.005)

We next examined if STEP_61_ mediates the TTX- or BC-induced changes in Tyr-phosphorylation of GluN2B and GluA2. Transactivator of transcription (TAT) sequence was fused to STEP_46_ and a myc tag (Fig. 4a), allowing the TAT fusion proteins to be membrane permeable (Additional file 1: Figure S1A, B) [15]. Preincubation for 30 min with active TAT-STEP wild-type (WT) but not control inactive TAT-myc reduced the levels of GluN2B-pY^1472^ and GluA2-p3Y in CTL-treated neurons (Fig. 4b, c, Additional file 1: Figure S1C) and occluded the TTX-induced increase in GluN2B-pY^1472^ and GluA2-p3Y levels compared to CTL application (Fig. 4b, c), suggesting that the increase in Tyr-phosphorylation of GluN2B and GluA2 is mediated by the TTX-induced reduction in STEP_61_.

In TAT-STEP C/S, a C300S point mutation inactivates STEP_46_, allowing it to bind constitutively to substrates but not to dephosphorylate them [13, 15, 16]. Consistently, introduction of TAT-STEP C/S in CTL-treated neurons significantly increased the levels of GluN2B-pY^1472^ and GluA2-p3Y compared to TAT-myc application (Fig. 4d, e, Additional file 1: Figure S1D). Preincubation with TAT-STEP C/S but not TAT-myc blocked the BC-induced reduction in the levels of GluN2B-pY^1472^, total GluN2B, and total GluA2 but not GluA2-p3Y (Fig. 4d, e). Since specific Tyr residues regulated by STEP_61_ remain unknown, our analyses for GluA2-p3Y may not have revealed the effect of TAT-STEP C/S if STEP_61_ causes dephosphorylation of only one Tyr. Nonetheless, these results suggest that STEP_61_ mediates the BC-induced changes in Tyr^1472^-phosphorylation of GluN2B and abundance of GluN2B and GluA2.

### Enhancement of STEP activity blocks synaptic scaling

**Fig. 5:**
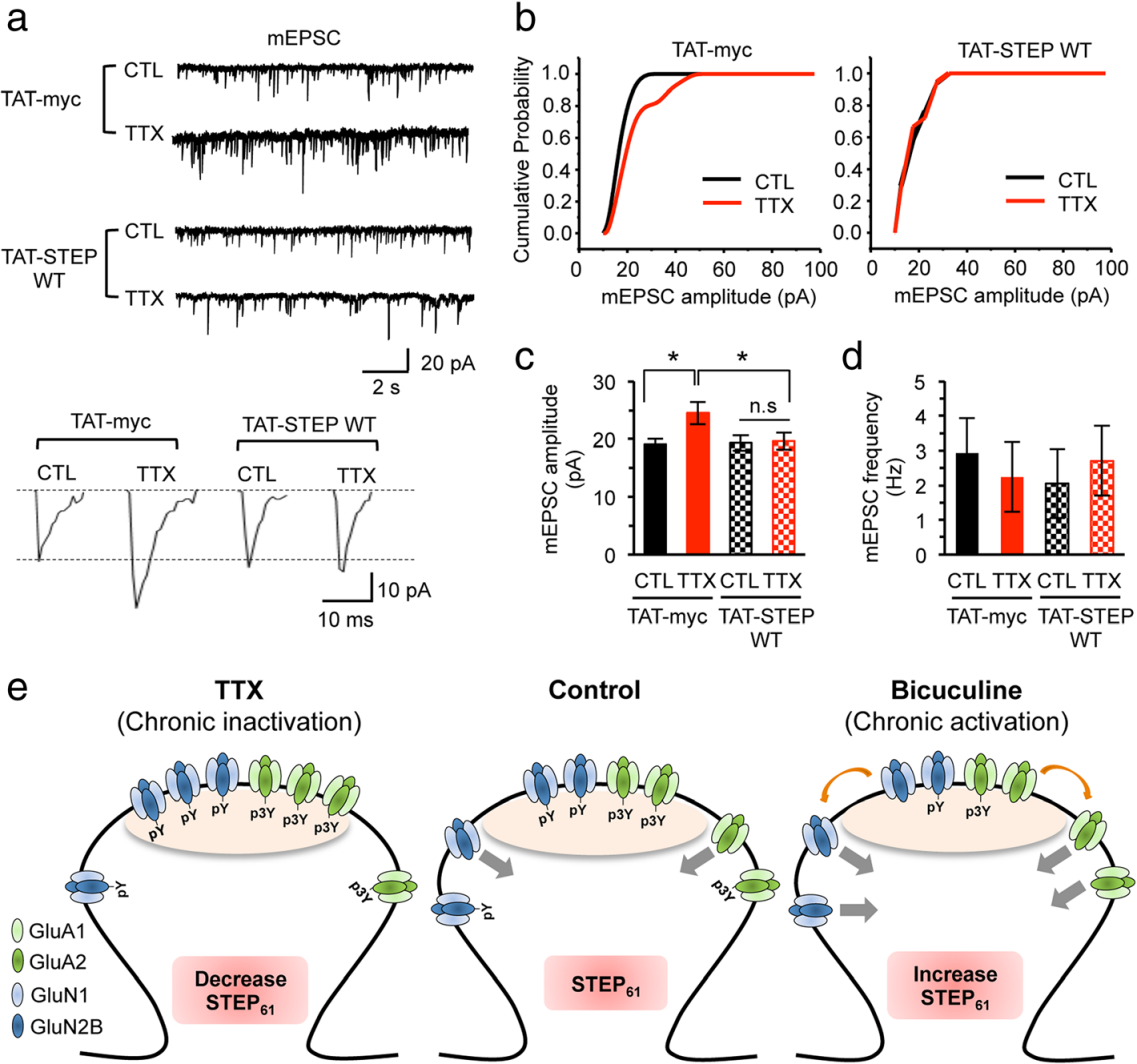
TAT-STEP WT blocks synaptic scaling induced by prolonged inhibition of hippocampal network activity. **a**–**d** Whole-cell patch clamp recording of mEPSCs from cultured hippocampal neurons that were treated for 48 h with vehicle control (CTL, 0.1 % H_2_O) and TTX (1 μM). Prior to mEPSCs recording, neurons were preincubated for 30 min with TAT-myc and TAT-STEP WT protein. **a** Representative traces of mEPSCs. **b** Normalized cumulative fraction of the mEPSC amplitudes. **c** TAT-STEP WT abolished the TTX-induced increase in the mEPSC amplitudes. **d** TAT-STEP WT did not affect the mEPSC frequencies. Data shown (**c**, **d**) represent the mean ± SEM (*p < 0.05). **e** Model by which activity-dependent changes in STEP_61_ level and activity regulate Tyr-dephosphorylation of GluA2 and GluN2B, leading to changes in surface AMPAR and NMDAR expression during homeostatic synaptic plasticity. Gray arrows indicate internalization. Orange arrows indicate lateral movement of surface AMPAR and NMDAR out of postsynaptic density (light pink)

Dephosphorylation of Tyr^1472^ within a conserved endocytic motif of GluN2B [17] via STEP_61_ reduces surface NMDAR level [4, 6] by clathrin-mediated internalization [18]. Furthermore, AMPAR internalization can be induced by mGluR stimulation through STEP_61_ [3] and by dephosphorylation of GluA2 at 3Tyr [14]. We hypothesized that prolonged activity blockade induces synaptic scaling (Fig. 1a–d) by inhibiting endocytosis of synaptic NMDARs and AMPARs upon STEP_61_ reduction (Fig. 1f, Fig 2b). To test this, we enhanced STEP activity by administering TAT-STEP WT for 30 min prior to recording. In the presence of TAT-myc, 48 h TTX treatment increased the mEPSC amplitude but not frequency compared to CTL application (Fig. 5a–d). However, this TTX-induced synaptic scaling was abolished by TAT-STEP WT preincubation (Fig. 5a–d), indicating that STEP_61_ reduction contributes to synaptic scaling.

Interestingly, prolonged activity enhancement increased STEP_61_ (Fig. 1g, Fig. 2c) and decreased Tyr-phosphorylation of GluN2B and GluA2 (Fig. 3d–f, Fig. 4d, e) without inducing synaptic down-scaling (Fig. 1a–d), suggesting that this STEP_61_ upregulation may cause internalization of extrasynaptic GluN2B and GluA2. Indeed, activity-dependent AMPAR endocytosis requires GluA2 [19] and occurs extrasynaptically [20]. Similarly, GluN2B-containing NMDARs enriched in extrasynaptic sites [21] undergo robust endocytosis [17, 18]. The BC-induced STEP_61_-dependent decrease in GluA2 and GluN2B abundance (Fig. 3d, e, Fig. 4d, e) may provide an additional homeostatic defense to limit membrane depolarization and overstimulation of extrasynaptic GluN2B-containing NMDARs, which is shown to cause excitotoxicity [22].

It remains unknown how prolonged activity perturbation regulates STEP_61_. Previous studies have reported that Ser^221^ of STEP_61_ is dephosphorylated by calcium-dependent calcineurin upon NMDAR activation [13] and phosphorylated by protein kinase A (PKA) upon stimulation of dopamine D1 receptor [23]. Interestingly, synaptic scaling is shown to involve reduced calcium influx to the postsynaptic neuron [9], reduced calcineurin activity [24], and enhanced PKA activity at excitatory synapses [25]. Hence, prolonged activity blockade could increase Ser^221^-phosphorylation of STEP_61_ (Fig. 2b, d) by reduced calcineurin activity and/or enhanced PKA activity, in addition to decreasing STEP_61_ level by transcriptional down-regulation (Fig. 1e, f). Considering that a loss of PKA from synapses was found during synaptic downscaling [25], reduced PKA activity may contribute to the BC-induced decrease in Ser^221^-phosphorylation of STEP_61_ (Fig. 2c, d).

## Conclusions

In summary, we demonstrate a bidirectional modulation of STEP_61_ level and activity by prolonged alterations of hippocampal network activity, resulting in correlative changes in Tyr-phosphorylation of STEP_61_ substrates, GluN2B and GluA2. We also show that the reduction in STEP_61_ contributes to synaptic scaling. Future studies should test if this regulation alters NMDAR and AMPAR surface density during homeostatic plasticity (Fig. 5e). Investigating how prolonged activity perturbation regulates STEP_61_ should provide mechanistic insights into the dysregulation of STEP_61_ expression, which are present in multiple neuropathologies [2].

## Materials and methods

### Hippocampal neuronal culture

The Institutional Animal Care and Use Committee at the University of Illinois Urbana-Champaign approved all experimental procedures involving animals. Primary dissociated hippocampal cultures were prepared from Sprague–Dawley rat embryos at embryonic day 18 and plated at high density (330 cells/mm^2^) as described [8]. At 10–13 days in vitro, neurons were treated for 24–48 h with vehicle control (0.1 % dH_2_O), TTX (1 μM), and BC (20 μM) (all Tocris).

### Electrophysiology

Whole-cell patch-clamp recordings of mEPSCs (>150 events per neuron) were performed at 23–25 °C from pyramidal neurons held at −60 mV in external solution containing 1 μM TTX and 20 μM BC as described [12, 25] using a Multiclamp 700B amplifier, Digidata1440A, and the pClamp 10.2 (Molecular Devices). Signals were acquired 3 min after making the whole-cell configuration, filtered at 1 kHz, and sampled at 10 kHz on gap free mode (5 min). The mEPSCs were detected with a 10 pA thresholds and analyzed by Mini Analysis (Synaptosoft).

### QPCR

The QPCR was performed with the StepOnePlus real-time PCR system (Applied Biosystems) using total RNA (1–2 μg) as described [8]. The forward and reverse primer sequences for *PTPN5* were 5’-GGAGTCAGCCCATGAATACC-3’ and 5’-CAGACGTACCCTGCTGTGAG-3’ respectively. The primer sequences for *GAPDH* has been previously described [8]. Following normalization to control *GAPDH* cDNA levels, the fold change of *PTPN5* cDNA levels for each treatment compared to control was determined.

### Immunoblot analysis

Neuronal lysate samples were prepared in RIPA buffer supplemented with protease inhibitors and Tyr phosphatase inhibitors (1 mM NaVO_3_, 10 mM Na_4_O_7_P_2_, and 50 mM NaF) as described [8] and were subjected to immunoblot analysis with primary antibodies against STEP_61_ (Santa Cruz), STEP_61_-pS^221^([13]), GluN2B and GluA2 (Millipore), GluN2B-pY^1472^ (PhosphoSolutions), GluA2-p3Y and GAPDH (Cell Signaling). Densitometric quantification following normalization to GAPDH was performed with ImageJ software (National Institutes of Health).

### Immunocytochemistry

Permeabilized immunostaining were performed with anti-myc antibodies (Thermo-Scientific) as described [12, 25]. Fluorescence images of the neurons were acquired using the same exposure time and analyzed with ImageJ to compare their background-subtracted fluorescence intensities.

### Statistical analyses

Using Origin 9.1 (Origin Lab), the Student’s *t* test and one-way ANOVA with Tukey’s and Fisher’s multiple comparison tests were performed to identify the statistically significant difference with a priori value (*p*) < 0.05 between 2 groups and for >3 groups, respectively.

## Additional file

Additional file 1: Figure S1. Membrane-permeable TAT-STEP WT or C/S proteins alter STEP_61_–dependent Tyr-phosphorylation of GluN2B and GluA2. (A) Permeabilized immunostaining of cultured hippocampal neurons at 12 days in vitro that were incubated for 30 min with no fusion proteins (None), TAT-myc, TAT-STEP WT, or TAT-STEP C/S. Scale bars are 20 μm. (B) Background subtracted, mean intensity of myc fluorescence (*n* = 10–19 images per treatment). AU, arbitrary unit. (C–D) Quantification of the levels of Tyr^1472^–phosphorylated GluN2B (GluN2B-pY^1472^) and the level of GluA2 that were phosphorylated at Tyr ^869^, Tyr ^873^, and Tyr ^876^ (GluA2-p3Y) in CTL-treated neurons (from Fig. 4b–e) that were incubated with TAT-fusion proteins for 30 min. (C) TAT-STEP WT decreases basal Tyr-phosphorylation of GluN2B and GluA2, confirming that TAT-STEP WT increases STEP activity. (D) TAT-STEP C/S increases basal Tyr-phosphorylation of GluN2B and GluA2, confirming its ability to block dephosphorylation of STEP substrates. Data shown represent the mean ± SEM (**p* < 0.05; ***p* < 0.01). (PDF 969 kb)

## Abbreviations

STEP: STriatal-Enriched protein tyrosine Phosphatase
Ser: Serine
Tyr: Tyrosine
AMPAR: α-amino-3-hydroxyle-5-methyl-4-isoxazolepropionic acid receptor
NMDAR: *N*-methyl-D-aspartate receptor
CTL: Control
TTX: Tetrodotoxin
BC: Bicuculline
PKA: Protein kinase A
mEPSC: miniature excitatory postsynaptic current
QPCR: real-time quantitative polymerase chain reaction
