# Supplementary figures and images for: Regulation of STEP61 and tyrosine-phosphorylation of NMDA and AMPA receptors during homeostatic synaptic plasticity
Source: Mol Brain. 2015 Sep 22;8:55. doi: 10.1186/s13041-015-0148-4 (PMC4578242; doi:10.1186/s13041-015-0148-4)

**A**

None

TAT-myc

TAT-STEP WT

TAT-STEP C/S

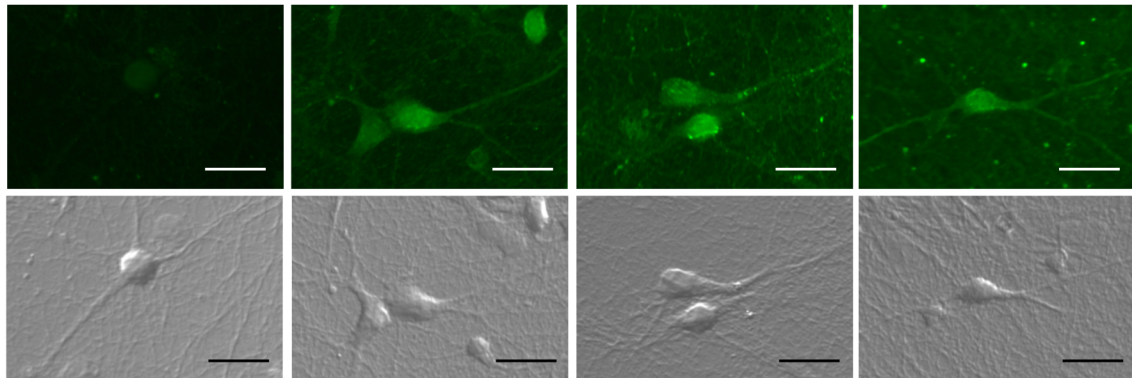**B**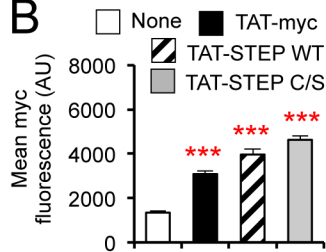**C**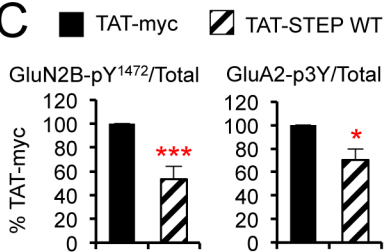**D**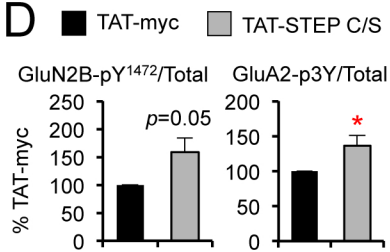

Supplement: Additional file 1: Figure S1. — Membrane-permeable TAT-STEP WT or C/S proteins alter STEP61–dependent Tyr-phosphorylation of GluN2B and GluA2. (A) Permeabilized immunostaining of cultured hippocampal neurons at 12 days in vitro that were incubated for 30 min with no fusion proteins (None), TAT-myc, TAT-STEP WT, or TAT-STEP C/S. Scale bars are 20 μm. (B) Background subtracted, mean intensity of myc fluorescence (n = 10–19 images per treatment). AU, arbitrary unit. (C–D) Quantification of the levels of Tyr1472–phosphorylated GluN2B (GluN2B-pY1472) and the level of GluA2 that were phosphorylated at Tyr 869, Tyr 873, and Tyr 876 (GluA2-p3Y) in CTL-treated neurons (from Fig. 4b–e) that were incubated with TAT-fusion proteins for 30 min. (C) TAT-STEP WT decreases basal Tyr-phosphorylation of GluN2B and GluA2, confirming that TAT-STEP WT increases STEP activity. (D) TAT-STEP C/S increases basal Tyr-phosphorylation of GluN2B and GluA2, confirming its ability to block dephosphorylation of STEP substrates. Data shown represent the mean ± SEM (*p < 0.05; **p < 0.01). (PDF 969 kb) [file 13041_2015_148_MOESM1_ESM.pdf]
